# Modeling tree crown dynamics with 3D partial differential equations

**DOI:** 10.3389/fpls.2014.00329

**Published:** 2014-07-21

**Authors:** Robert Beyer, Véronique Letort, Paul-Henry Cournède

**Affiliations:** Ecole Centrale Paris, Applied Mathematics and Systems LaboratoryChâtenay-Malabry, France

**Keywords:** leaf area density, continuity equation, functional-structural plant model, crown plasticity, competition for light, Beer-Lambert's law

## Abstract

We characterize a tree's spatial foliage distribution by the *local leaf area density*. Considering this spatially continuous variable allows to describe the spatiotemporal evolution of the tree crown by means of 3D partial differential equations. These offer a framework to rigorously take locally and adaptively acting effects into account, notably the growth toward light. Biomass production through photosynthesis and the allocation to foliage and wood are readily included in this model framework. The system of equations stands out due to its inherent dynamic property of self-organization and spontaneous adaptation, generating complex behavior from even only a few parameters. The density-based approach yields spatially structured tree crowns without relying on detailed geometry. We present the methodological fundamentals of such a modeling approach and discuss further prospects and applications.

## 1. Introduction

In terms of model scale, light sensitive functional-structural tree growth modeling has experienced the emergence of various trends. Organ-level approaches bring about a high precision of physiological processes, averting inaccuracies and effects of scale non-invariance, which may arise from simplifications in larger-scale approaches. Moreover, arbitrary small-scale biophysical or biochemical processes can in principle be readily induced. The LIGNUM model (Perttunen et al., 1996; Sievänen et al., 2008), the model by Sterck et al. (2005) or the L-Peach model (Allen et al., 2005) are examples of this model category. Local light interception (cf. also Chelle and Andrieu, 2007 for a methods review) determines the production and allocation of biomass. Their detail of physiological and morphological processes is at the same time the drawback of these models. On the one hand, the large number of organs implies high computational costs—all the more in competition scenarios of multiple trees. On the other hand, their detail can make these models susceptible to the propagation of errors, which could have been compensated for in an averaging rough scale approach.

Other organ-level models like Greenlab (Yan et al., 2004; Cournède et al., 2006) a priori focus on the topology in terms of the plant's structure. This implies the inability of easily taking physiological and structural responses to varying local light conditions into account. As one consequence, the approach cannot be straightforwardly applied to scenarios of competition for light: An additional yet non local competition index provides for this (Cournède et al., 2008). In another context of formal grammars used for tree growth simulation, Kurth and Sloboda (1999) present the 2D concept of the shadow-relevant cone of a shoot in order to take local light conditions into account, which in turn affect the rewriting system. Comparable methods have been used by Purves et al. (2007) and Takenaka (1994).

Sonntag (1996) presents a model in which the spatial motion and allocation of leaf area is based on heuristic rules on a 2D cellular space. Though quite different in terms of formalism, his approach bears conceptual resemblances to ours.

Models with a rougher scale use to impose certain characteristics on the crown shape. For instance, the Balance model Grote and Pretzsch (2002) and the model by Sorrensen-Cothern et al. (1993) describe the crown shape in terms of disk-like horizontal layers. This technique implies advantages with regard to the computational speed as well as a general robustness, compared to small-scale models. Yet it does so at the expense of a thorough plastic spatial crown structure. When applied to competition scenarios, these models often make use of empirically fitted competition indices (e.g., Pretzsch, 1992).

While being affiliated more with the latter forestry models in the attempt to describe crown structure and dynamics macroscopically for applications at the stand level, the present approach attempts a middle course between the fine organ-centered way and the rougher pre-imposition of the crown form. We characterize a tree's spatial foliage distribution via its *local leaf area density*. The focusing on this variable circumvents difficulties in terms of robustness in the geometrically detailed models accounting for individual leaf positions. At the same time, the locality allows for arbitrary spatial structures.

Applying Beer Lambert's law allows to express the local light conditions within a crown as a function of the local leaf area density. Aiming at an increased future light interception, local leaf area density is assumed to tend to move toward the light. This approach, which induces the spatial expansion of the crown, translates directly into a partial differential equation. Details are specified based on mass conservation and optimization considerations. The technique notably allows to account for a local and spontaneous adaptiveness with regard to changing environmental light conditions.

The mathematical approach of density-based partial differential equations has long established itself in spatial biology and ecology (cf. e.g., Okubo and Levin, 2002). In the context of macroscopic individual plant modeling, so far notably in the form of diffusion equations, it has proven applicable for root growth and proliferation (see Page and Gerwitz, 1974 for the original approach, Reddy and Pachepsky, 2001 for a review of later developments and Dupuy et al., 2010 for a current advance). A 2D diffusion approach for the foliage of crops with the objective to model competition in different field densities, without considering the vertical dimension, was presented by Beyer et al. (2014). Partial differential equations can generate, even from only a few terms, complex self-adapting dynamics, which is indeed present in biological systems. Attempts to reproduce this by simpler terms often requires a larger model framework and set of parameters. The latter, in turn, requires large data sets, which are often not available.

In this article we will exemplify the use of the leaf area density-based partial differential equation approach by means of a simplified model setup presented in section 2. The continuity of the approach suggests embedding into in the context of continuously growing trees sensu (Hallé et al., 1978), i.e., “with no marked endogenous cessation of extension [and] a more or less constant production of leaves and/or shoots throughout the year” (Barthélémy and Caraglio, 2007).

Since merely selected dynamics are presented, without constructing a realistic model, we settle for illustrations of key qualitative properties of the approach instead of quantitative data comparison. Throughout the article, we will point out possibilities of extending and customizing process assumptions while preserving the advantages of the overall methodological framework. Extending future steps are discussed in section 3.

## 2. Model framework

For clarity's sake all recurrently appearing variables and parameters and their definitions are listed in Table 1. We describe the spatial foliage distribution of a tree canopy by means of the *(local) leaf area density* α(*x, t*) ≥ 0 (in m^2^ m^−3^) in a point *x* = (*x*_1_, *x*_2_, *x*_3_) ∈ ℝ^3^ (each entry in m) at a time *t* ≥ 0 (in years), i.e., the spatial density of the total one-sided green leaf area (previously considered e.g., by Sinoquet et al., 2005). The map α(·, *t*): ℝ^3^ → ℝ, *x* ↦ α(*x, t*) is continuous for any *t*. For brevity, we will use the notion *leaf density* in place of leaf area density.

**Table 1:** **Key model variables and parameters**.

α(*x, t*)	Leaf area density in *x* at time *t* (in m^2^ m^−3^)
α(·, *t*)	The map ℝ^3^ → ℝ, *x* ↦ α(*x, t*)
Λ	Mean leaf transmittance
λ(*x, v*)	Cosine of the angle between leaf plane normal and sun ray
*S*^2^_+_	Upper half unit sphere {*x* ∈ ℝ^3^:‖*x*‖ = 1, *x*_3_ ≥ 0}
μ	Energetic efficiency (in g MJ^−1^)
*PAR*(*v, t*)	Radiation from direction *v* at time *t* (in MJ m^−2^ s^−1^)
*b*(*x, t*)	Local biomass production in *x* at time *t* (in g s^−1^)
*SLA*	Specific leaf area (in m^2^ g^−1^)
*WD*	Wood density (in g m^−3^)
*P*	Pipe model theory constant (in m^2^ m^−2^): 1 unit $\alpha \hat{=}P$ *P* Units pipe cross-sectional area
‖*x*‖_ϒ_	Length of the sapwood pipe leading to *x* (in m)
*L*(*x, t*)	Local radiation in *x* induced by the leaf density α(*t*) (in MJ m^−2^)

We aim to describe the evolution *t* ↦ α(·, *t*). To this end we will first determine the biomass production *B* of a tree, which, along with the senescence *S* of old biomass, allows to describe the net biomass increment of a tree corresponding to a leaf density α(·, *t*) at a given time *t*:

$$\frac{\partial }{\partial t}m(t)=B(t)-S(t)$$

This, in turn, will be distributed among foliage and sapwood according to the pipe model theory (Shinozaki et al., 1964), specified in the subsequent paragraph. The coaction of foliage allocation and senescence in the case of a continuously growing tree induces what we abstractly interpret as a continuous motion of α(·, *t*), in particular directed toward the light, aiming at an increased future biomass production. This perspective leads to the description of the course of *t* ↦ α(·, *t*) essentially by means of a continuity equation.

*Sapwood Associated to a Leaf Density:* The pipe model theory by Shinozaki et al. (1964) allows to determine the sapwood mass corresponding to an arbitrary leaf density α(·, *t*). In the present context, the theory states that for any point *x* ∈ ℝ^3^ with α(*x, t*) > 0, a sapwood pipe, in charge of the transport of water and nutrients, leads from *x* down to the roots with a length denoted by ‖*x*‖_ϒ_, its cross-sectional area being proportional to the leaf density α(*x, t*) at its tip via a constant *P*. The mass of the pipe leading to *x* then equals

$$\underset{\text{cross-sectional area}}{\underset{︸}{\alpha (x,t)\cdot P}}\cdot \;\underset{\text{length}}{\underset{︸}{\|x{\|}_{\Upsilon }}}\cdot \;WD,$$

*WD* denoting the wood density (in g m^−3^).

It is worth mentioning that, motivated by the limitations to the pipe model theory, pointed out e.g., by Tyree (1988), Pouderoux et al. (2001), and Deleuze and Houllier (2002), generalizations have been suggested: A noteworthy approach is the one by Bouchon et al. (1997), who, based on an allocation perspective, reason that the pipe does not necessarily have a constant cross-sectional area along its path, but more generally a one exponentially decreasing toward the stem base. This principle integrates in our context with only minor technical changes. Likewise, the approach by Letort et al. (2008), parametrically combining the pipe model approach and a uniform, common pool sapwood allocation, would be feasible.

As for the pipe's length ‖*x*‖_ϒ_ from *x* to the root tip, for the sake of simplicity, here we follow the multi-species approach used by Sonntag (1996), based on the branch architecture of coniferous species and the assumption that root length equals branch length, resulting in

(1) $$\|x{\|}_{\Upsilon }\;:=\;|\;x_{3}|+2\;\cdot \;\|(x_{1},x_{2})\|\;.$$

A more accurate choice for a particular species can be made by taking specific characteristics of its branching geometry (such as branching angles) and topology into account.

Finally, the sum of foliage and sapwood mass is given by

(2) $$\underset{\text{foliage mass}}{\underset{︸}{\int_{{\mathbb{R}}^{3}}\frac{\alpha (x,t)}{SLA}\;dx}}+\underset{\text{sapwood mass}}{\underset{︸}{\int_{{\mathbb{R}}^{3}}\alpha (x,t)\cdot P\cdot \|x{\|}_{\Upsilon }\cdot \;WD\;dx}}$$

where *SLA* denotes the specific leaf area (in m^2^ g^−1^).

### 2.1. Biomass production

We determine the amount of biomass produced through photosynthetic activity by a given leaf density. To this, we take direct and diffuse radiation into account, cf. Fu and Rich (1999), using the horizontal celestial coordinate system, with ℝ^2^ × {0} being the local horizon of a tree rooting in (0, 0, 0), and the vector (1, 0, 0) pointing north. The unit directional vector σ(*t*) ∈ *S*^2^_+_: = {*x* ∈ ℝ^3^: ‖*x*‖ = 1, *x*_3_ ≥ 0}, under which the sun is seen from the tree at daytime *t* reads σ(*t*) = (cos(−Az(*t*)) · cos(Alt(*t*)), sin(−Az(*t*)(*t*)) · cos(Alt(*t*)), sin(Alt(*t*))), where Alt(*t*) and Az(*t*) denote the time dependent altitude and azimuth, respectively.

For diffusive radiation, a uniform diffuse model (uniform overcast sky) is applied, in which incoming diffuse radiation is assumed to be the same from all sky directions. Let *PAR*_dir_(*t*) and *PAR*_diff_(*t*) (in MJ m^-2^) denote the photosynthetically active direct and diffuse radiation at time *t*, respectively. Then the total radiation from direction *v*∈ *S*^2^_+_ at time *t* is

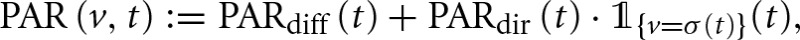



with the indicator function 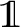
_*A*_(*x*) : = 1 if *x* ∈ *A* and 0 else.

#### 2.1.1. Isolated tree

Incoming radiation is partly intercepted by the tree's foliage and partly passes through it. The fraction between 0 and 100% of radiation from direction *v*∈ *S*^2^_+_ which actually reaches the point *x* ∈ ℝ^3^ can be determined using Beer-Lambert's law, where foliage characterized by leaf density acts as a light absorbing medium with locally varying α-concentration. This fraction reads

(3) $$\exp\text{​}(-\Lambda \cdot \int_{x+{\mathbb{R}}_{+}\cdot v}\lambda (\xi ,v)\cdot \alpha (\xi ,t)d\xi )$$

where the extinction coefficient Λ ≤ 1 represents the mean light transmittance of foliage (Monteith, 1969; Nouvellon et al., 2000) and

λ(x,v):=N(x)·v

takes into account the angle between the sun ray and foliage in *x*, *N*(*x*) ∈ *S*^2^_+_ denoting the unit normal to the plane in which foliage in *x* lies. *N*(*x*) can be chosen according to leaf angle distribution models without further ado (Wang et al., 2007) provide a review.

Assuming that local biomass production is proportional to the total locally intercepted ratiation via an energy efficiency μ (in g MJ^−1^) (Monteith, 1977), the instantaneous local biomass production *b*(*x, t*) (in g m^−3^ s^−1^) in *x* ∈ ℝ^3^ at time *t* for the leaf density α(·, *t*) reads

(4) $$\begin{array}{l} b(x,t)=\mu \cdot \\ \int_{S_{+}^{2}}\underset{\text{radiation from direction }v\text{ intercepted in }x}{\underset{︸}{\lambda (x,v)\cdot \alpha (x,t)\cdot \underset{\text{radiation reaching }x\text{ from direction }v}{\underset{︸}{\exp(-\Lambda \cdot \int_{x+{\mathbb{R}}_{+}\cdot v}\lambda (\xi ,v)\cdot \alpha (\xi ,t)d\xi )\cdot \text{PAR}(v,t)}}}}dv.\;\;\;\;\;\; \end{array}$$

#### 2.1.2. Population of trees

At a time *t*, let α_1_(·, *t*), …, α_*n*_(·, *t*) denote the leaf densities of *n* trees, shading each other and competing for light. Then the instantaneous local biomass production of tree *i* ∈ {1, …, *n*} in *x* generalizes to

$$\begin{array}{l} b_{i}(x,t)=\mu \cdot \int_{S_{+}^{2}}\;\frac{{\left(\lambda_{i}(x,v)\cdot \alpha_{i}(x,t)\right)}^{2}}{\sum_{j=1}^{n}\lambda_{j}(x,v)\cdot \alpha_{j}(x,t)}\cdot \\ \;\exp\left(-\Lambda \cdot \int_{x+{\mathbb{R}}_{+}\cdot v}\sum_{j=1}^{n}\lambda_{j}(\xi ,v)\cdot \alpha_{j}(\xi ,t)\;d\xi \right)\text{​}\cdot \text{​PAR​}(v,t)dv \end{array}$$

The fraction $\frac{\lambda_{i}(x,v)\cdot \alpha_{i}(x,t)}{\sum_{j=1}^{n}\lambda_{j}(x,v)\cdot \alpha_{j}(x,t)}$ is the part of the incoming radiation in *x* that is attributed to tree *i*'s foliage in *x*. In particular it reduces to 1 if two trees' crowns do not occupy common space.

### 2.2. Dynamics

#### 2.2.1. Mass balance

We determine the instantaneous change in living mass (conductive sapwood and foliage mass) due to the production of new, and the senescence of old biomass. For convenience we assume that the senescence of leaves as well as the loss of conductivity of sapwood depend on time only. If sapwood and foliage that have existed for τ_*W*_ and τ_*F*_ years become nonconductive and senescent, respectively, then the living mass at time *t* reduces by

$$\begin{array}{l} S(t):=\int_{{\mathbb{R}}^{3}}\frac{\frac{\partial }{\partial t}\alpha (x,t-\tau_{F})}{SLA}dx+\int_{{\mathbb{R}}^{3}}\frac{\partial }{\partial t}\alpha (x,t-\tau_{W})\cdot \\ \;P\cdot \|x{\|}_{\Upsilon }\cdot WD\;dx. \end{array}$$

At the same time it increases by the total instantaneous biomass production at *t*, i.e., *B*(*t*) : = ∫_ℝ^3^_ *b*(*x, t*)*dx*. Thus we have

(5) $$\frac{\partial }{\partial t}m(t)=B(t)-S(t)$$

for the change in living mass at time *t*. A priori, the possibility $\frac{\partial }{\partial t}m(t)<0$ is not excluded for arbitrary parameters. However, since *m*(*t*) > *S(t)*, it follows that *m*(*t*) > 0 and thus *B*(*t*) > 0 for all *t* ≥ 0.

#### 2.2.2. Leaf density dynamics

With this information on the global mass of the tree at hand, we consider the variable

$$\hat{\alpha }(x,t)=\frac{\alpha (x,t)}{\int_{{\mathbb{R}}^{3}}\alpha (x,t)dx},$$

i.e., the leaf density modulo mass, or *(mass-)relative* leaf density, with the property that ∫_ℝ^3^_$\hat{\alpha }$(*x, t*)*dx* = 1 for all *t*, which is understood as an indicator of the spatial structure of the real leaf density α(·, *t*). Instead of describing the course of α(·, *t*) directly, we do so for $\hat{\alpha }$(·, *t*) and deduce α(·, *t*) as α(·, *t*) = λ(*t*) · $\hat{\alpha }$(·, *t*)}, where λ(*t*) is chosen such that the living mass corresponding to λ(*t*) · $\hat{\alpha }$(·, *t*) (cf. (2)) equals indeed *m*(*t*) yielded by (5). Thus

(6) $$\lambda (t)=\frac{m(t)}{\int_{{\mathbb{R}}^{3}}\frac{\hat{\alpha }(x,t)}{SLA}\;dx+\int_{{\mathbb{R}}^{3}}\hat{\alpha }(x,t)\cdot P\cdot \|x{\|}_{\Upsilon }\cdot WD\;dx}.$$

The continuity of the growth process of the trees we consider suggests a description of the course of $\hat{\alpha }$(·, *t*) in terms of a (mass-conserving) continuity equation,

(7) $$\frac{\partial }{\partial t}\hat{\alpha }(x,t)=\nabla_{x}\cdot \phi (x,t),$$

in which the relative leaf density is subject to a transport motion induced by a continuous flux ϕ: ℝ^3^ × ℝ_+_ → ℝ^3^, itself determined by α(·, *t*). The idea of this transporting flux ϕ is that it incorporates both the effect of the allocation of new leaves and of the abscission of old ones on the spatial structure of foliage, which in combination, induces what we describe as an abstract motion of leaf density.

A predominant driver in the spatial dispersal of leaf density is the local expansion toward the light, aiming at an increase in future light interception. We formally embed this factor in the above framework, where it will take on the role of the flux ϕ. For some given leaf density α(·, *t*) let *L*: ℝ^3^ × ℝ_+_ → ℝ be defined by

(8) $$\begin{array}{l} L(x,t)=\int_{S_{+}^{2}}\lambda (x,v)\;\cdot \;\exp(-\Lambda \cdot \int_{x+{\mathbb{R}}_{+}\cdot v}\lambda (\xi ,v)\cdot \alpha (\xi ,t)d\xi )\cdot \\ \text{ PAR}(v,t)\;dv \end{array}$$

The function *L* measures the intercepted light in *x* per m^2^ leaf area. The gradient ∇_*x*_*L* points locally in the direction of the greatest rate of increase of intercepted light. In addition, similar to Beyer et al. (2014) we define the flux to correspond to the existing leaf density in *x*, so that finally we have

(9) $$\phi (x,t)=k\cdot \alpha (x,t)\cdot \nabla_{x}L(x,t)$$

for a mobility constant *k*. This local gradient approach is motivated by “the observation that a tree is capable of acquiring also gradient information about its environment and that growth might be directed along these gradients (Schmidt and Wulff, 1993; Aphalo and Ballare, 1995)” (Sonntag, 1996).

The term ∇_*x*_ · ϕ describing a movement of leaf density toward the light contains spatial derivates of a function of integrals over α, which makes (7) a partial integro-differential equation.

### 2.3. Simulations

In this section we illustrate some structural properties of the model. For convenience we simplified radiation to be vertical only. Some details of the numerical implementation of the model are presented in the appendix.

**Figure 1:**
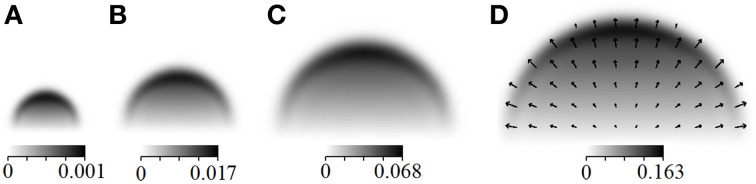
**(*x*_1_, *x*_3_)-cross section of a leaf density-characterized crown in the course of time, subject to the present model dynamics, corresponding to *t* = 10 **(A)**, 20 **(B)**, 30 **(C)** and 40 **(D)** years with simulation parameters adopted from Letort et al. (2008)**. A darker color indicates a higher leaf density α. The arrows in **(D)** indicate the vector field ϕ.

**Figure 2:**
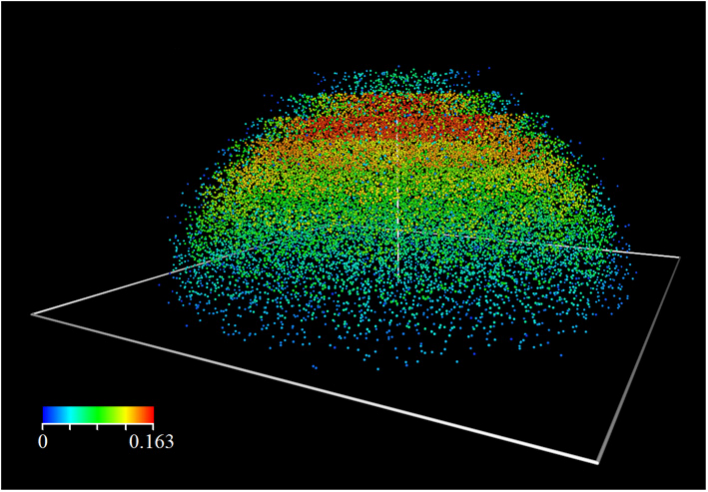
**3D view of Figure 1D**.

Figures 1, 2 illustrate the evolution of leaf density in the course of time, as well as the vector field ϕ. The term is sensitive to any change in the local light conditions induced by shading; ϕ instantaneously adapts and points in the direction of the greatest light increase. Aside the general spatial expansion toward the light, we notably observe the predominant presence of foliage at the crown hull rather than its interior.

#### 2.3.1. Population of trees

Together with the adjustments in terms of biomass production addressed at the end of section 2.1, the approach generalizes to competition scenarios when α(·, *t*) is replaced by ∑_*i* ≥ 1_ α_*i*_(·, *t*) in (8), alongside the different initial states α_1_(·, 0), α_2_(·, 0), …. We illustrate the dynamic effects to which competition gives rise by means of a simplified scenario: Consider a sufficiently large stand, in which the trees' stem bases, as a point set in ℝ^2^ × {0}, generate a Voronoi-tesselation which is regular. If radiation is assumed to be radially symmetric (as done e.g., by Perttunen et al., 1998), the analysis of all competing trees reduces to that of a single one for which periodic boundary conditions for (7) are added on the boundary of the tree's 3D cell, i.e., the extension of the appropriate 2D Voronoi cell in the *x*_3_-dimension.

Periodic boundary conditions induce that the light conditions on the other side of the boundary are considered identical to those within, accounting for another tree growing in equal measure and shading its environment.

This implies that, when, for simplicity, further assuming the essential light incidence to be vertical, periodic boundary conditions reduce to no flux conditions on the boundary: There, due to the identical light conditions on the other side of the boundary, the light gradient ∇_*x*_*L*, governing the flux ϕ, changes from pointing further outwards to a zero flux.

**Figure 3:**
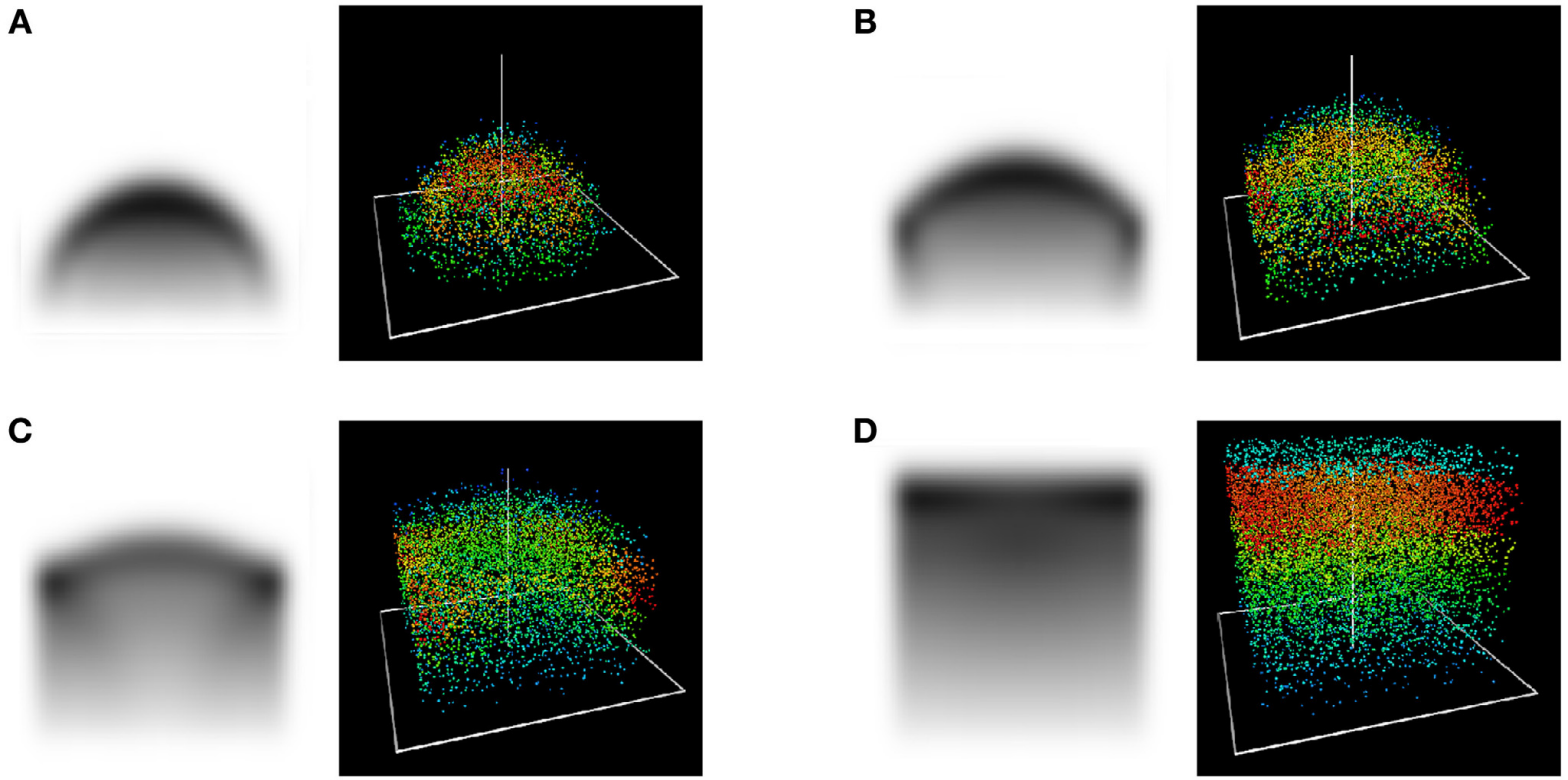
**(*x*_1_, *x*_3_)-cross section and 3D view of the stages in a competition scenario at *t* = 10 **(A)**, 20 **(B)**, 30 **(C)** and 40 **(D)** years with periodic bounday conditions at 3.5 m**. Due to properties of self-symmetry, visible in Figure 1, these stages are in fact qualitatively similar no matter the cell size.

Figure 3 shows the different stages of this scenario for an underlying square tesselation.

The overly sharp boundary between two crowns is a consequence of the unrestrained mobility of foliage in this simplified approach, and would fade when additional features corresponding to a branch structure are included, cf. section 3.

The conspicuous concentration of leaf density at the upper edges of the canopy in the competition case (cf. Figure 3C) results from the two factors of (i) the regular expansion of leaf density uninfluenced by the boundary condition and (ii) the immigration of leaf density from lower regions whose horizontal expansion had abruptly turned into a vertical one after reaching the Voronoi cell boundary. We are unable to provide evidence or counterevidence to determine whether this phenomenon corresponds to reality. In any case, as observable in Figure 3D, this is only an intermediate state, before in the long-term a homogeneously distributed high concentration of leaf density in the top canopy establishes itself.

**Figure 4:**
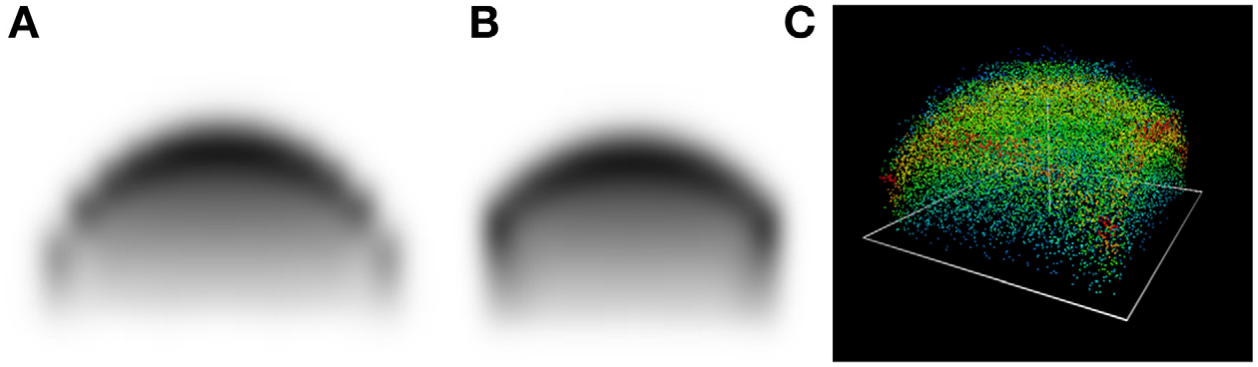
**Cross-section of (A) a diagonal plane (B) the (*x*_1_, *x*_3_)-plane at *t* = 15 years. (C) 3D view**.

Wheras an isolated tree grows radially symmetric, this symmetry is eventually broken for a non-circular Voronoi cell, such as the square used just now. Figure 4 illustrates this.

## 3. Discussion and future prospects

The aim of this article was to show how local leaf area density, a concept opposing geometrically detailed individual leaf configurations, can be used to approach macroscopic tree crown dynamics. Its integration into the formal framework of partial differential equations allowed to rigorously formulate the growth toward light. In the simulations we observed the generation of self-organization and adaptiveness that come along with this modeling approach. The simplistic model framework was meant to draw attention to the key mechanisms and their dynamic effects.

Foliage dynamics are by nature coupled to the tree's branch structure, which has not been taken into account on a topological or geometric level in this article. Future work, with the aim of introducing more spatial heterogeneity to the approach, begins here. Taking merely the stem and the most vigorous primary branches into account while leaving the finer structures to the leaf area density concept may already suffice to tackle crown plasticity satisfactorily. While in the present simplified model the motion of local leaf area density is governed by light only and otherwise unrestrained, a simple branch architecture can add sort of directional inertia to that motion, channeling local leaf area density in several major directions, resulting in heterogeneous foliage clustering, representing individual branches. In particular, this includes the incorporation of genetically predetermined branching angle spectra. Introducing a branch structure, even if only a rough one, is moreover accompanied by a refinement of the pipe length term (1), governing the distribution of mass between foliage and wood according to the pipe model theory, thus determining secondary growth.

Taking into account the organization of growth units, i.e., as weakening our assumptions on neoformation and polycyclism in section 2.1.1, or considering immediate vs. delayed bud outbreak, would bring the model closer to actual tree architecture dynamics.

Alongside the phototropism considered in this article, more biomechanical constraints that have feedback influences on the growth, such as hydraulic aspects sensu (Ryan and Yoder, 1997; Tyree and Zimmermann, 2002), in particular in the context of growth limitation, the avoidance of interlocked growth due to mechanical stress of touching branches (Oliver and Larson, 1990) or gravitropism represent perspectives for model extensions.

The application and validation of a refined model based on the theoretical framework presented in this paper will benefit from empirical data on local leaf area density. Conceivable ways to obtain this include the following three, which are currently being practically explored: Firstly, from the direct recordings of local light intensities at various positions {*x*^(1)^, …, *x*^(*n*)^} ⊂ ℝ^3^ within a canopy, the map

$$\begin{array}{l} \{x^{\left(1\right)},\ldots ,x^{\left(n\right)}\}\to {\mathbb{R}}_{\geq 0}\\ \;x\;\mapsto \alpha (x) \end{array}$$

for a discrete, but arbitrary fine domain can be obtained by applying Beer-Lambert's law in the reverse way. Secondly, high-definition multi-directional 3D terrestrial laser scan data and appropriate skeletonization algorithms allow to relate to a leafless tree a set of cylinders representing branch segments (Raumonen et al., 2013). Automatically removing all scan points corresponding to cylinder diameters (i.e., branch thicknesses) above a certain threshold allows to spatially isolate recent shoots, for which then a relation to foliage can be assumed. Thirdly, scanning a tree at the beginning of spring during the process of bud opening, when—in contrast to the case of a fully developed canopy—laser rays still reasonably penetrate the crown, and deducting from this image the point cloud yielded by a scan of the completely leafless tree, may allow to obtain a local bud density, from which the the leaf density can be deduced. Thus, assessing the spatial foliage distribution in terms of local leaf area density in a functional-structural crown dynamics model can make good use of this data type for model calibration and validation.

The property of locally and spontaneously adapting to changing light conditions suggests that the present partial differential equation approach can be applied to competition scenarios both in pure as in mixed tree groups. Empirical findings, based on laser scans, about the plasticity Bayer et al. (2013) may thus be approached from a functional-structural modeling point of view. These perspectives are currently being explored.

### Conflict of interest statement

The authors declare that the research was conducted in the absence of any commercial or financial relationships that could be construed as a potential conflict of interest.

## 4. Appendix

### Numerical implementation

A finite volume scheme Toro (2009) is used, in which we consider ${\bar{\alpha }}_{ijk}:=\frac{1}{\Delta^{2}}\int_{\Sigma_{ijk}}\alpha (x,t)dx$ (with a similar notation for the other variables) on a regular mesh with cells Σ_*ijk*_ = [*i*Δ*x*, (*i* + 1) Δ*x*[× [*j*Δ*x*,(*j* + 1) Δ*x*[×[*k*Δ*x*,(*k* + 1)Δ*x*] for *i, j, k*∈ ℤ and Δ*x* « 1.

The local light incidence (8) (and similarly the local biomass production (4)) for vertical radiation, *v* = *e*_3_, is computed as

$$\begin{array}{l} {\bar{L}}_{ijk}(t)={\bar{\lambda }}_{ijk}(e_{3})\cdot \exp(-\Lambda \cdot \Delta x\cdot \sum_{\kappa \geq k}{\bar{\lambda }}_{ijk}(e_{3})\cdot {\bar{\alpha }}_{ijk}(t)\cdot \\ \text{ PAR}(e_{3},t)dv) \end{array}$$

The general case for *v*∈ *S*^2^_+_ is conceptually similar, yet technically more extensive: For a given *v*, the above sum reaches over all cells Σ_*i*'*j*'*k*'_ whose intersection with the line through the center of the cell Σ_*i*'*j*'*k*'_ and pointing in the direction *v* is non-empty, taking into accout the individual length ℓ_*i*′*j*′*k*′_(*v*) ≤ Δ*x* of this intersection by means of the additional coefficient $\frac{\ell_{i^{\prime }j^{\prime }k^{\prime }}(v)}{\Delta x}$ in the sum term.

As for the PDE, let

$${\bar{\phi }}_{1,ijk}(t)=k\cdot \frac{{\bar{\alpha }}_{i+1jk}(t)+{\bar{\alpha }}_{ijk}(t)}{2}\cdot \frac{{\bar{L}}_{i+1jk}(t)-{\bar{L}}_{ijk}(t)}{\Delta x}\;$$

and ϕ_2,*ijk*_, ϕ_3,*ijk*_ be defined accordingly for the other spatial entries. The standard discretization of the divergence is given by

$$\begin{array}{l} {\bar{\text{div}}}_{ijk}(t)=\frac{{\bar{\phi }}_{1,ijk}(t)-{\bar{\phi }}_{1,i-1jk}(t)}{\Delta x}+\frac{{\bar{\phi }}_{2,ijk}(t))-{\bar{\phi }}_{2,ij-1k}(t)}{\Delta x}\\ \;+\frac{{\bar{\phi }}_{3,ijk}(t))-{\bar{\phi }}_{3,ijk-1}(t))}{\Delta x} \end{array}$$

and finally, using the Euler method for Δ*t* « 1, we obtain

$${\bar{\alpha }}_{ijk}(t+\Delta t)={\bar{\alpha }}_{ijk}(t)+\Delta t\cdot {\bar{\text{div}}}_{ijk}(t).$$
